# A DNA methylation age predictor for zebrafish

**DOI:** 10.18632/aging.202400

**Published:** 2020-12-23

**Authors:** Benjamin Mayne, Darren Korbie, Lisa Kenchington, Ben Ezzy, Oliver Berry, Simon Jarman

**Affiliations:** 1 Environomics Future Science Platform, Indian Ocean Marine Research Centre, Commonwealth Scientific and Industrial Research Organisation (CSIRO), Crawley, Western Australia, Australia; 2 Australian Institute for Bioengineering and Nanotechnology (AIBN), The University of Queensland, St Lucia, Queensland, Australia; 3 Western Australian Zebrafish Experimental Research Centre (WAZERC), University of Western Australia, Perth, Western Australia, Australia; 4 School of Biological Sciences, The University of Western Australia, Perth, Western Australia, Australia

**Keywords:** age estimation, CpG sites, DNA methylation, multiplex PCR, sequencing

## Abstract

Changes in DNA methylation at specific CpG sites have been used to build predictive models to estimate animal age, predominantly in mammals. Little testing for this effect has been conducted in other vertebrate groups, such as bony fish, the largest vertebrate class. The development of most age-predictive models has relied on a genome-wide sequencing method to obtain a DNA methylation level, which makes it costly to deploy as an assay to estimate age in many samples. Here, we have generated a reduced representation bisulfite sequencing data set of caudal fin tissue from a model fish species, zebrafish (*Danio rerio*), aged from 11.9-60.1 weeks. We identified changes in methylation at specific CpG sites that correlated strongly with increasing age. Using an optimised unique set of 26 CpG sites we developed a multiplex PCR assay that predicts age with an average median absolute error rate of 3.2 weeks in zebrafish between 10.9-78.1 weeks of age. We also demonstrate the use of multiplex PCR as an efficient quantitative approach to measure DNA methylation for the use of age estimation. This study highlights the potential further use of DNA methylation as an age estimation method in non-mammalian vertebrate species.

## INTRODUCTION

Ageing on a cellular level involves the decline of molecular functions resulting in a cumulative decrease in cell maintenance [1]. Biological ageing at the organism level is at least partly driven by molecular and cellular changes [2]. Biological ageing is observed in most animal species [3, 4] and several epigenetic changes occur during the ageing process [5, 6]. DNA methylation at cytosine-phosphate-guanine (CpG) sites is known to change with age.

A growing number of studies have shown that changes in DNA methylation at a set of specific CpG sites are predictive of age [7]. These studies have constructed what is often referred to as epigenetic clocks and can be used to predict chronological or biological age [8–11]. Epigenetic clocks have predominantly been derived for mammalian species but has also been developed for a sea bird (*Ardenna tenuirostris*) [7, 12]. There has been little exploration of whether DNA methylation can be used for age estimation in other vertebrate groups. The identification of DNA methylation biomarkers for age estimation has typically relied on data-intensive reduced representation bisulfite sequencing (RRBS) or Illumina Infinium microarrays [13–16]. While effective for marker identification and model development, these methods are not well-suited to high-throughput characterisation of age for large numbers of non-human samples. RRBS generates a high volume of data that makes it computationally expensive. Microarrays have not been developed for most species outside of humans and model organisms such as mice because of the large expense involved in development.

Age is fundamental information in fisheries as it can be used to estimate abundance [17], sustainable harvests, and population growth rates [18]. In short lived species the length of the fish is often used as a substitute for age [19, 20] otherwise otoliths (ear bones) are used for most species [21–23]. It is possible to obtain daily increments by otoliths for short lived fish [24]. However, this becomes difficult for fish greater than 10 years old, where only annual increments can be measured [25]. DNA methylation-based age-estimation may offer a robust alternative in fishes, and further, provide sub-annual age increments. Age estimation from otoliths is also lethal as it involves the removal of the inner ear bone. Non-lethal age estimation methods are ethically preferable and compatible with multiple live capture survey methods such as genetic capture-recapture studies [26]. In this study, we use zebrafish as a model species to develop a cost-effective approach for age estimation in fish. Zebrafish are an ideal species for this work as individuals with known ages are readily available.

Zebrafish are a short-lived species that can reproduce at 10 weeks and with an average lifespan in captivity of 150 weeks [27]. This short life cycle, their regenerative ability, and their senescent phenotype with increasing age makes them a valuable model for vertebrate ageing research [28]. Zebrafish are highly fertile and are generally inexpensive to maintain making them an ideal model species for experimental research on any aspect of fish biology [29]. Moreover, zebrafish have a long history as a genetic model organism, with a well-characterised genome that can be genetically manipulated [30–32]. The zebrafish epigenome experiences variation due to environmental factors similar to other species [33]. DNA methylation also has a pivotal role in zebrafish embryonic development orchestrating the transcriptome and regulating cell development [33, 34]. However, it is unknown if DNA methylation is predictive of age during post-embryonic development, as seen in mammals [7, 35]. Here, using RRBS and a known age series we identify DNA methylation biomarkers that accurately predict the age of zebrafish from caudal fin tissue. We develop a multiplex PCR assay for the affordable and efficient measurement of DNA methylation to estimate age with high accuracy and precision.

## RESULTS

### Age marker identification by reduced representation bisulfite sequencing

Full details on the maintenance of the zebrafish colony can be found in Supplementary File 1. On average, 45.1 million reads per RRBS library (Supplementary File 1 Data) was aligned to the zebrafish genome with an alignment rate of 87.4%. This resulted in a total of 524,038 CpG sites with adequate coverage in at least 90% of all samples (see Methods). Of these sites, 60.9% were within gene bodies such as exons (Supplementary Figure 1). Global CpG methylation level was on average 79.5%, similar to what has been observed in other zebrafish tissues [36–38]. We found no correlation between global CpG methylation and age (Pearson correlation = 0.030, p-value = 0.77). However, we identified methylation at 1,311 CpG sites to significantly correlate (p-value < 0.05) with increasing age, similar to what has been found in mice [15]. This suggests specific CpG sites are associated with ageing but not epigenetic drift as indicated by global methylation [39].

**Figure 1:**
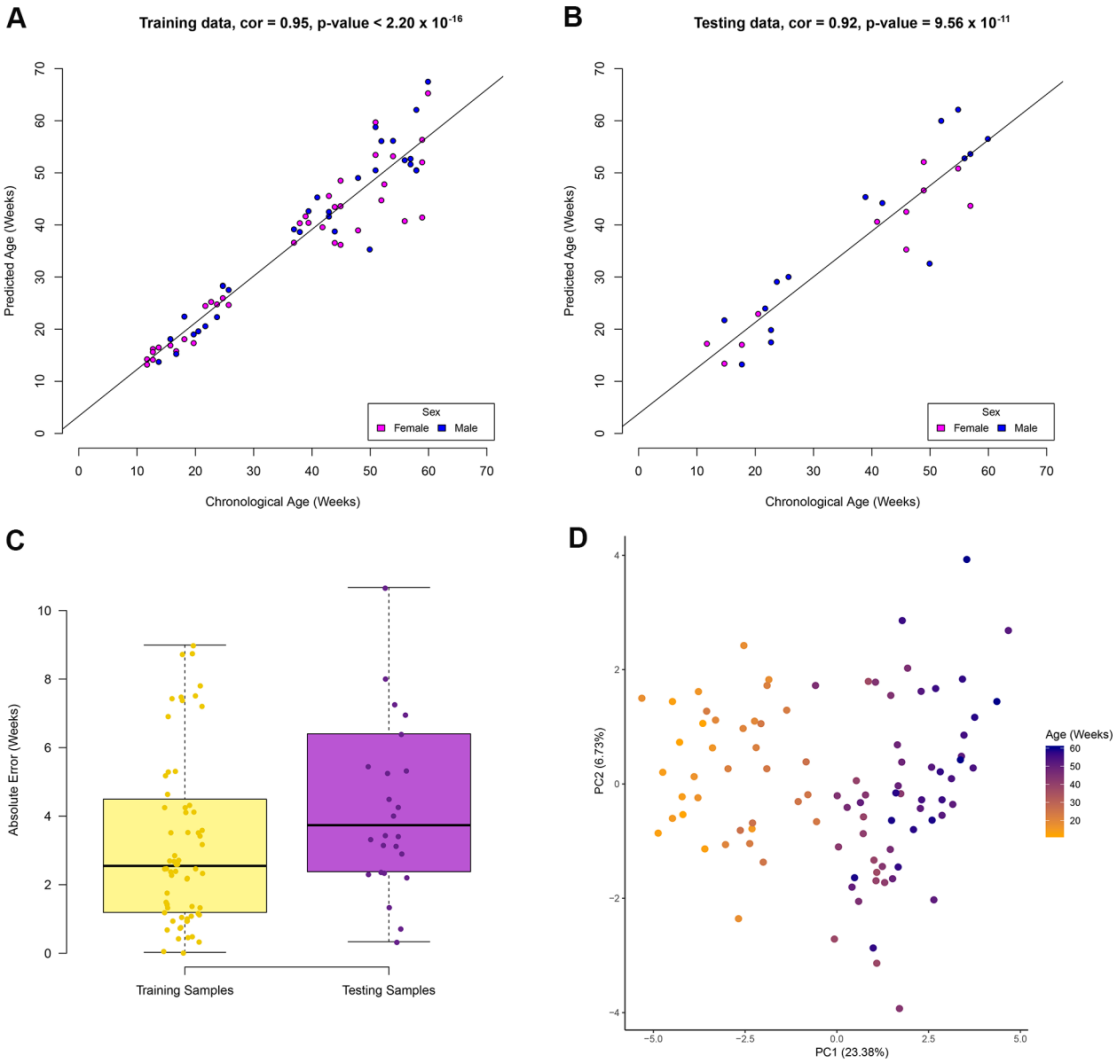
**Zebrafish age estimation from DNA methylation of 29 CpG sites.** Performance of the model in the (**A**) training data set, (**B**) testing data set. Colour represents the sample sex in the correlation plots. (**C**) Boxplots show the absolute error rate in the training and testing data sets. (**D**) Unsupervised clustering of samples using the 29 CpG sites show separation based on age in the first principle component.

An elastic net regression model was used to regress age over the 70% of the RRBS samples (67 samples). The regression model returns the minimum number of sites required to estimate age (see Methods). Our model to estimate age in zebrafish using RRBS data was based on a total of 29 CpG sites (Supplementary Table 2). In the training data set a high correlation (Pearson correlation = 0.95, p-value < 2.20 x 10^-16^) between the chronological and predicted age was observed (Figure 1A). In addition, a high correlation (Pearson correlation = 0.92, p-value = 9.56 x 10^-11^) between these variables in the testing data set was also observed (Figure 1B). A median absolute error (MAE) rate of 3.7 weeks was found in the testing data set (Figure 1C) and no statistical difference was observed between the absolute error rate between the training and testing data sets (p-value = 0.14, t-test, two-tailed). The similar performance between the training and testing data sets suggests a low possibility of overfitting. A PCA was used to visualise the separation of samples by age using the methylation levels of the 29 CpG sites (Figure 1D). The first principal component explains 23.4% of the variation by age. This unsupervised clustering shows separation of the samples solely on increasing age, suggesting the 29 CpG sites are ideal candidates to estimate age. Samples did not cluster by sex suggesting the age associated sites are not sex specific (Supplementary Figure 2). No significant gene ontology (GO) enrichment was observed for the 29 CpG sites using Enrichr. Using a zebrafish background set of genes, four genes (*meis2a, gnptab, hoxb3a, mab21l2*) relating to embryonic skeletal system morphogenesis were identified (adjusted p-value = 0.06). It should be noted that this is not a significant GO result. However, it is a similar pattern to what has been observed in age associated CpG sites in humans as many are related to embryonic development [14]. From here on the 29 CpG sites will be referred to as the zebrafish clock sites.

### Multiplex PCR followed by sequencing

**Figure 2:**
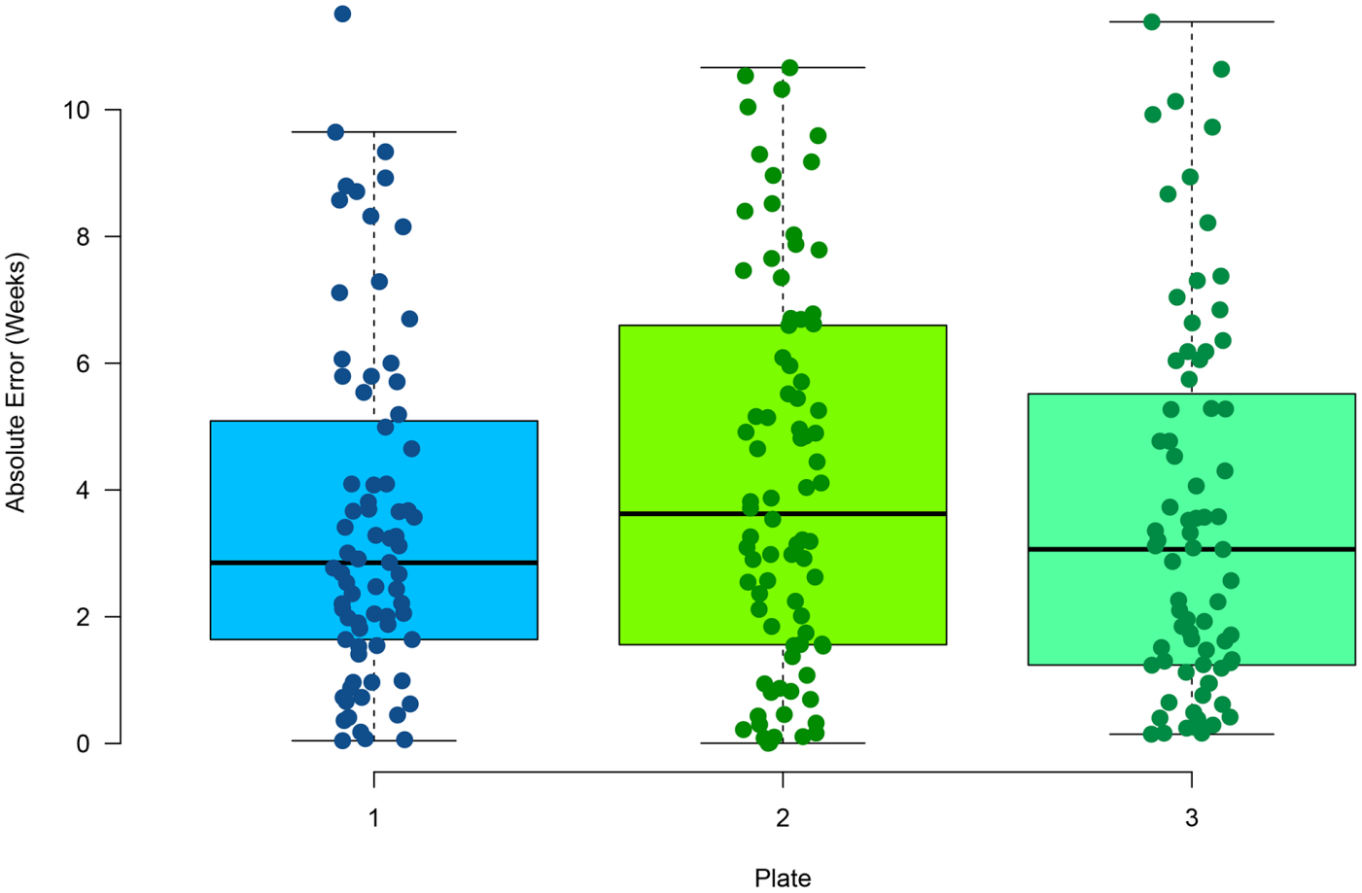
**Performance of age estimation by multiplex PCR showing the absolute error rate for the 96 samples in triplicate.**

Multiplex PCR has been used previously to measure DNA methylation at select CpG sites [40]. In comparison to RRBS, it provides a 100-fold decrease in sequencing cost, making a more cost-effective approach for targeted sequencing. For each zebrafish clock CpG site, primers were designed to amplify an approximate 140bp amplicon inclusive of the CpG site (Supplementary Table 3). Three CpG sites were removed from the model due to lack of amplification by primers (see Methods). Caudal fin tissue from 96 zebrafish that were not part of the initial RRBS and aged between 10.9 - 78.1 weeks was used to test the multiplex PCR assay. Samples were assayed in triplicate to determine reproducibility of the method. On average, 459,000 reads were aligned to the reference genome, with 15,248 reads per amplicon, with an alignment rate of 98.5%. By having a saturation of high read coverage per amplicon reduces any potential of read variation on methylation levels. We found a high average correlation across the replicates (Supplementary Figure 3) between the chronological and predicted age (Pearson correlation = 0.97) and a low average (mean) MAE of 3.18 weeks (Figure 2). No statistically significant difference was found between the absolute error rates between replicates (p-value = 0.366, one-way ANOVA), suggesting the method is highly reproducible. In addition, no statistically significant difference was found between the absolute error rate in the RRBS testing data set and the multiplex PCR samples (p-value = 0.23, t-test, two-tailed). This suggests RRBS and multiplex PCR return similar sensitivities in methylation values. A median relative error of 8.2% was also observed in the multiplex PCR assay. No significant difference was found between the residuals and increasing age (Supplementary Figure 4) suggesting a consistent relative error rate with age.

### Methylation-sensitive PCR

Methylation sensitive PCR (msPCR) can be deployed as a rapid and cost-effective method to assay methylation of selected CpG sites, but has not been used to quantify methylation for the estimation of age [41]. We used msPCR as a potential alternative non-sequencing methodology to quantify DNA methylation for age prediction. Primers (Supplementary Table 4) were designed to target the zebrafish clock sites (see Methods). Despite a significant correlation between the chronological and predicted age (Pearson correlation = 0.62, p-value = 0.00028) the MAE rate increased 261% (13.4 weeks) compared to the RRBS MAE rate (Supplementary Figure 5). This analysis suggests msPCR is not sensitive enough as the relative error was 36.2%.

### Epigenetic drift

**Figure 3:**
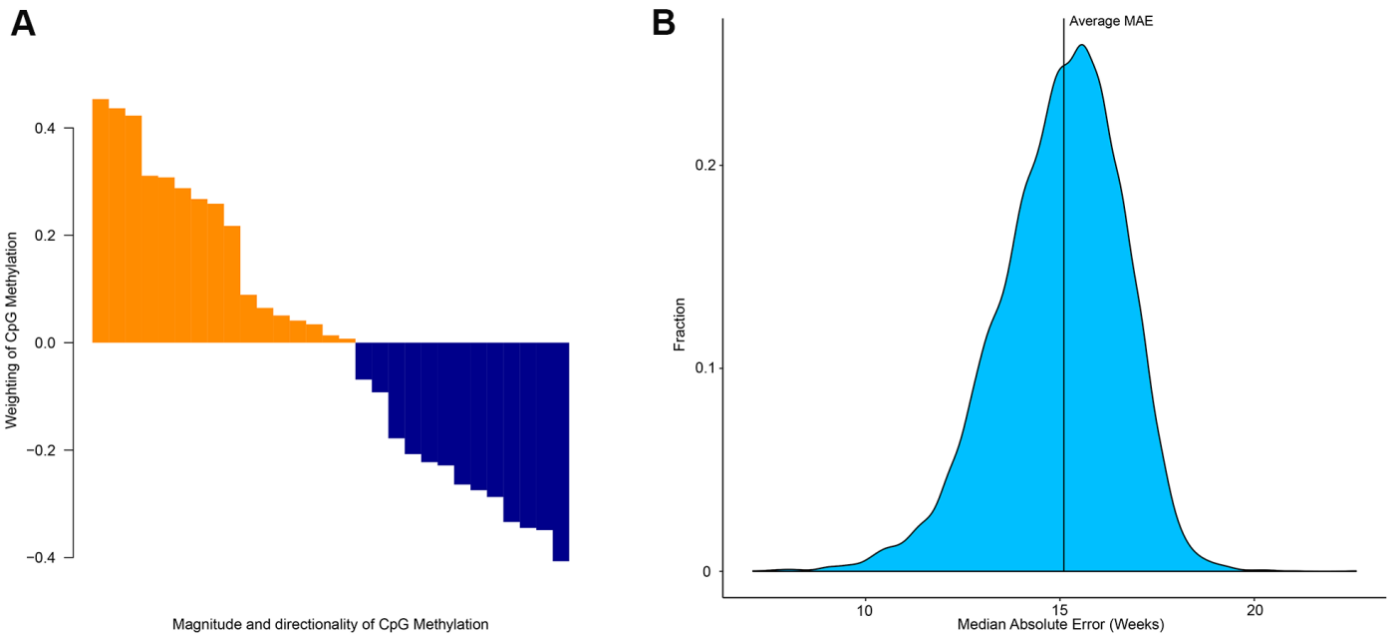
**Importance of specific CpG sites in estimating the age of zebrafish.** (**A**) Weighting and directionality of each of the 29 CpG age associated sites. (**B**) Distribution of the performance of 10,000 age-estimation models in the form of median absolute error (weeks).

The elastic net regression model returns the minimum number of CpG sites required to estimate age. However, these sites differ in terms of importance for age prediction. Each CpG site has a different weight (Figure 3A), but collectively can be used to estimate age. This demonstrates that despite each CpG site having a different degree of age-association, collectively multiple CpG sites can be used to accurately estimate age [14]. To determine the level of age-association in other CpG sites we used a ridge model (α-parameter = 0 in glmnet) and randomly selected 29 CpG sites out of the possible 524,038 CpG sites. This was repeated 10,000 times and produced an average MAE of 15.1 weeks (Figure 3B). This analysis demonstrates that collectively CpG sites can be predictive of age, however certain CpG sites are better candidates as biomarkers of age.

## DISCUSSION

We have developed the first epigenetic clock for zebrafish. By developing a high-resolution DNA methylation map for zebrafish caudal fin tissue, we were able to identify age associated CpG sites that can be collectively used to estimate age. Previous studies have developed assays to target small numbers of CpG sites to estimate age [42, 43] or used data-intensive genome-wide approaches [15, 16]. This is the first study to both develop a genome-wide characterisation of age-associated CpG sites in zebrafish and to construct a high-throughput assay deployable in a basic molecular biology laboratory for specific CpG sites that estimate age.

The changes in DNA methylation at specific CpG sites with increasing age are generally small and it is rare for methylation levels to go from completely unmethylated to fully methylated or vice versa. Therefore, when measuring methylation at specific CpG sites it is essential for the assay to be highly sensitive. The efficacy of msPCR was investigated because of its modest technical requirements in comparison to DNA sequencing approaches. In other applications msPCR has delivered sufficient sensitivity to cancer prognosis assays [44]. However, msPCR was insufficiently sensitive for the detection of the small changes in methylation during ageing in zebrafish. In contrast, multiplex PCR appears to be an ideal cost-effective approach to estimate age from CpG methylation. As other age-estimation studies have shown, fewer than 110 CpG sites is required to estimate age [16, 42, 43]. Therefore, if estimating age is the only requirement, it is only necessary to perform sequencing on CpG sites that are predictive of age rather than the more data intensive and costly RRBS. Multiplex PCR followed by sequencing is an ideal tool to estimating age as it can focus on genomic regions of interest and still provide the same sensitivity as RRBS. This method for age estimation has an advantage over array-based methods for many researchers in that arrays are only available for a limited range of species and the technology for implementing array-based epigenetic clocks is not available in many laboratories.

The aim of the study was to demonstrate the use of a cost-effective method of age estimation in a model fish. However, just as similar clocks in mice and humans have been used, there is the potential for it also to be used as a proxy for zebrafish health [14, 15]. Known age profiles are key data used to inform stock assessments in harvested fishes, and existing methods to age fish is lethal and subject to error. There is the potential of applying the age associated CpG sites in zebrafish in other bony fish where there is the conservation of DNA sequence. This approach has been used with success in mammals [42, 43]. Unlike human and mice studies, which were multi-tissue, the zebrafish epigenetic clock is tissue specific, and therefore may not accurately predict age from methylation data collected from other tissues. Caudal fin was selected because it is a widely sampled in fisheries management context and can be collected non-lethally [45]. Establishing a clock for fish caudal fin is therefore a necessary proof of concept for application to other species.

The first age-predictive model for a fish was developed by [46], who used multiplex PCR to estimate the age of European Seabass (*Dicentrarchus labrax*). That study also produced a highly accurate model (Pearson correlation = 0.824, MAE= 2.149 years), based on targeting candidate genes involved in tissue specific development [46]. Our study took an approach that has previously been used to identify age biomarkers in mice and dogs [15, 16] and that is less dependent on prior knowledge. The zebrafish model produced a more accurate model than the European Seabass (Pearson correlation = 0.97). However, it’s unclear whether this reflects differences between the species, or the method used to identify predictive CpG sites. Regardless of the performance of the models, the addition of the zebrafish epigenetic clock demonstrates the possibility of DNA methylation being predictive of age in a wide variety of fish. Under ideal conditions zebrafish live up to an average of 182 weeks in captivity [27]. This study used zebrafish up to 78.1 weeks as older individuals were unavailable. It is therefore unknown how well the model will perform on older individuals. The model was also developed with one zebrafish strain (AB). In any type of machine learning, the model will generally perform best on data similar characteristics to what it was trained on. To establish the generality of our results, in future the zebrafish model we have developed should ideally be tested in a subset of known age older fish and from other strains, including wild caught fish. However, if applying this model to wild zebrafish or applying similar tests for other wild fish species calibrated with a preponderance of younger individual, individuals near the maximum lifespan are generally very rare in wild populations. For many population biology research questions, older individuals are grouped into a “plus class” representing a wide age range, but very small counts [47].

The stochastic accumulation of error in the epigenome is often described as epigenetic drift and is a fundamental part of the ageing process [48, 49]. Using biological clocks to measure epigenetic drift has been previously suggested [35, 50, 51]. We found methylation at all CpG sites captured by RRBS have some level of age association, which is similar to results observed in mice [15]. This supports the notion of epigenetic drift as a factor in why DNA methylation performs as a marker of age. Yet, while epigenetic drift occurs as a random process across the genome, some CpG sites are significantly better predictors of age, suggesting additional functional drivers of age-related methylation. In humans, 30% of age associated DNA methylation is tissue specific [52]. There is also evidence that age associated DNA methylation occurs in bivalent domains and Polycomb group promoters [52, 53]. This suggests that age associated DNA methylation occurs in conjunction with specific epigenetic and genomic features over and above signals of epigenetic drift [54]. This is yet to be explored in non-human organisms such as zebrafish. Developing a better functional understanding of the mechanisms underpinning epigenetic clocks would enable more targeted identification of age biomarkers, especially in non-model organisms.

One of the limitations of using RRBS as it does not assess all CpG sites and more predictive sites may be missed. Furthermore, like all sequence-based approaches it can result in inconsistent coverage across sites, which may introduce error in the identification of age markers. Whole genome bisulfite sequencing would enable evaluation of all CpG sites, potentially revealing the strongest possible set of age predictive CpG sites. However, the high cost per sample and the large number of samples required to develop an age-estimation model would makes this a costly endeavour. Indeed, the high accuracy and precision demonstrated by our model developed from sites discovered through RRBS indicates that this approach suffices for developing accurate epigenetic clock for zebrafish. Similarly, the deep sequence coverage enabled by our PCR multiplex assay of CpG markers initially identified through RRBS shows that despite potential instances of low coverage in the marker identification phase, the biomarkers identified produce highly accurate estimates of zebrafish age. Microarrays represent an alternative means to assay CpG sites, with high accuracy and without suffering from low sequence coverage [55].

## CONCLUSIONS

This study is the first to develop a RRBS dataset for zebrafish caudal fin tissue from a broad range of ages and highlights the potential to use DNA methylation as a predictor of age in non-mammalian and non-avian animal groups. This is a valuable resource as it provides a time series of methylation in a species that is a model for development studies. Using this methylation data set we were able to identify CpG sites that collectively can be used to estimate age very accurately. Moreover, we were able to design a multiplex PCR assay to measure the methylation state at 26 CpG sites, at a significantly reduced cost and complexity of analysis compared to RRBS. Age has a central role in regulating the dynamics of animal populations and estimates of age-structure underpin almost all frameworks for wildlife and fisheries management. Yet, biomarkers for age are lacking for most animal groups. The transfer of epigenetic markers between mammal groups including humans, mice and bats indicates that similar approaches may be feasible in other groups such as fishes. The transfer of zebrafish epigenetic age markers to fishes with significant commercial or conservation importance would be of major significance considering the importance of age structure to management and the lack of effective non-lethal alternatives to estimating age.

## MATERIALS AND METHODS

### Zebrafish ageing colony

Zebrafish (AB strain) were bred and maintained at the Western Australian Zebrafish Experimental Research Centre (WAZERC). Refer to the Supplementary File 1 for full details on how the zebrafish were maintained. Animal ethics was approved by the University of Western Australia animal ethics committee (RA/3/100/1630). Animals aged between 10.9-78.1 weeks were euthanized using rapid cooling. Once deceased all organs and tissues were collected and stored into RNAlater (Thermo Fisher). DNA was extracted using the DNeasy Blood and Tissue Kit (QIAGEN) as instructed in the manufacturer’s protocol.

### Reduced representation bisulfite sequencing

A total of 96 RRBS libraries were prepared as previously described with digestion of the restriction enzyme *MspI* [56] at the Australian Genome Research Facility (AGRF) and were sequenced using an Illumina NovaSeq. Details of each zebrafish which were sequenced by RRBS are provided in Supplementary Table 1.

### Data availability

Raw sequencing data from RRBS has been made publicly available on the CSIRO Data Access Portal available at https://doi.org/10.25919/5f63ce026960a.

### RRBS data analysis

Fastq files were quality checked using FastQC v0.11.8 (https://www.bioinformatics.babraham.ac.uk/projects/fastqc/). Reads were trimmed using trimmomatic v 0.38 [57] with the following options: SE -phred33 ILLUMINACLIP:TruSeq3-SE:2:30:10 LEADING:3 TRAILING:3 SLIDINGWINDOW:4:15 MINLEN:36. Trimmed reads were aligned to the zebrafish genome (danRer10) using BS-Seeker2 v 2.0.3 default settings [58] and bowtie2 v2.3.4 [59]. Methylation calling was performed using BS-Seeker2 call methylation module with default settings. CpG sites were filtered out of the analysis if they had a mean coverage of < 2 reads or > 100 reads as what has occurred previously [15]. On average, each site per sample had a coverage of 16 reads.

### Predicting age from CpG methylation

Samples were randomly assigned to either a training (67 samples) or a testing data set (29 samples) using the createDataPartition function in the caret R package to maintain equal ratios of sex in each data set [60]. Age was transformed to natural log to fit a linear model. Using an elastic net regression model, the age of the zebrafish was regressed over all CpG site methylation in the training data set. Sites with missing data in less than 10% of samples were replaced with a methylation score of 0. By replacing the methylation score with 0 in samples with missing sites prevents any correction bias as the site will be removed from the analysis. The glmnet function in the glmnet R package [61] was set to a 10-fold cross validation with an α-parameter of 0.5 (optimal between a ridge and lasso model), which returned a minimum λ-value based on the training data of 0.02599415. These parameters resulted in a total of 29 CpG sites required to estimate the age of zebrafish. These 29 sites had methylation values in 100% of all samples. The performance of the model in the training and testing data set were assessed using Pearson correlations between the chronological and predicted age and the MAE rates.

### Principal component analysis and gene ontology

A principal component analysis (PCA) was used as a form of unsupervised clustering to visualise the age associated CpG sites in terms of separating samples by age. PCA was performed using FactoMineR [62]. Gene ontology (GO) enrichment was performed using the 2018 terms in the R package Enrichr and using the Generic Gene Ontology Term finder (https://go.princeton.edu/) [63, 64]. All analyses were performed in R using version 3.5.1 [65].

### DNA bisulfite conversion

DNA was bisulfite converted using the EZ DNA Methylation Gold Kit (Zymo Research) using manufacturer’s protocol or using a manual protocol as previously described [66].

### Multiplex PCR

A total of 96 independent zebrafish caudal fin tissue samples which were not part of the initial RRBS were used for multiplex PCR. Primers were designed using PrimerSuite [67] and were divided into two PCR reaction pools prior to barcoding (Supplementary Table 3). Three primer pairs were unable to be optimised as part of the overall multiplex PCR assay and were removed from the analysis. The remaining 26 CpG sites were remodelled using the RRBS methylation data by applying the ridge model component in the glmnet function (α-parameter = 0) resulting in alternative weights for each site (Supplementary Table 3). A generalised linear model was applied to the raw prediction values from the elastic net regression model (sum of the coefficient weights multiplied by the DNA methylation beta values). The final model to estimate age in zebrafish is:

ln(age)=1.008x

Where *x* is the sum of the raw summed methylation beta values for each sample.

Samples were run in triplicate to determine reproducibility of the method. The final 50μL PCR reaction contained 1x Green GoTaq Flexi Buffer (Promega), 0.025 U/μL of GoTaq Hot Start Polymerase (Promega), 4.5mM MgCl_2_ (Promega), 0.5x Combinatorial Enhancer Solution (CES) (Refer to [68]), 200μM of each dNTP (Fisher Biotec), 15mM Tetramethylammonium chloride (TMAC) (Sigma-Aldrich), primers (both forward and reverse) were used at 200nM and finally the bisulfite treated DNA (2ng/μL). Cycling conditions were 94° C/5mins; 12 cycles of 97° C/15 seconds and 45° C/30 seconds, 72° C/120 seconds; 1 cycle of 72° C/120 seconds and 6° C/hold. An Eppendorf ProS 384 thermocycler was used for amplification. Primers were designed using PrimerSuite [67] and primer sequences are provided in Supplementary Table 3.

### Barcoding

Oligonucleotides with attached MiSeq adaptors and barcodes were used for the barcoding reaction (Fluidigm PN FLD-100-3771). Barcoding was performed using 1x Green GoTaq Flexi Buffer, 0.05 U/μL of GoTaq Hot Start Polymerase, 0.25x CES, 4.5mM MgCl_2_, 200μM of each dNTP, 25μL of the pooled template after Sera-Mag Magnetic SpeedBeads (GE Healthcare Life Sciences) clean up. Cycling conditions for barcoding were as follows 94° C/5mins; 9 cycles of 97° C/15 seconds, 60° C/30 seconds and 72° C/2mins; 72° C/2mins; 6° C/5mins. Barcoding was performed using an Eppendorf ProS 96 or 384 thermocycler. Sequencing was performed on an Illumina MiSeq using the MiSeq Reagent Kit v2 (300 cycle; PN MS-102-2002).

### Multiplex PCR followed by sequencing data analysis

Sequencing data was hard clipped by 15bp at both 5′ and 3′ ends to remove adaptor sequences by SeqKit v 1.2 [69]. Reads were aligned to a reduced representation of the genome focusing on a 500bp upstream and downstream of the zebrafish clock sites. Reads were aligned using Bismark v 0.20.0 with the following options: --bowtie2 -N 1 -L 15 --bam -p 2 --score L,-0.6,-0.6 --non_directional and methylation calling was performed using bismark_methylation_extractor [70].

### Methylation sensitive PCR

msPCR primers were designed using MethPrimer v2.0 [71] which produces two pairs of primers for when the DNA is methylated and unmethylated. msPCR was optimised using the protocol detailed previously [44] with the final cycling conditions: Initialisation step 95° C/15 mins, denaturation step 95° C/30 seconds, annealing 55° C/40 seconds and extension 72° C/40 seconds, for 40 cycles. msPCR was performed using an AllTaq Mastermix (Qiagen) with 1 x SYBR Green (Thermo Fisher) in a Bio-Rad CFX96. The ΔCt values for each primer pair was used as a quantitative method for methylation. A leave-one-out cross validation approach was used to determine the level of precision for using msPCR to estimate age [60, 72].

## Supplementary Material

Supplementary Figures

Supplementary Table 1

Supplementary Table 2

Supplementary Table 3

Supplementary Table 4

Supplementary File 1
