# Supplementary material for: A DNA methylation age predictor for zebrafish
Source: Aging (Albany NY). 2020 Dec 23;12(24):24817–35. doi: 10.18632/aging.202400 (PMC7803548; doi:10.18632/aging.202400)
Supplement: Supplementary Figures [file aging-12-202400-s001.pdf]

SUPPLEMENTARY FIGURES

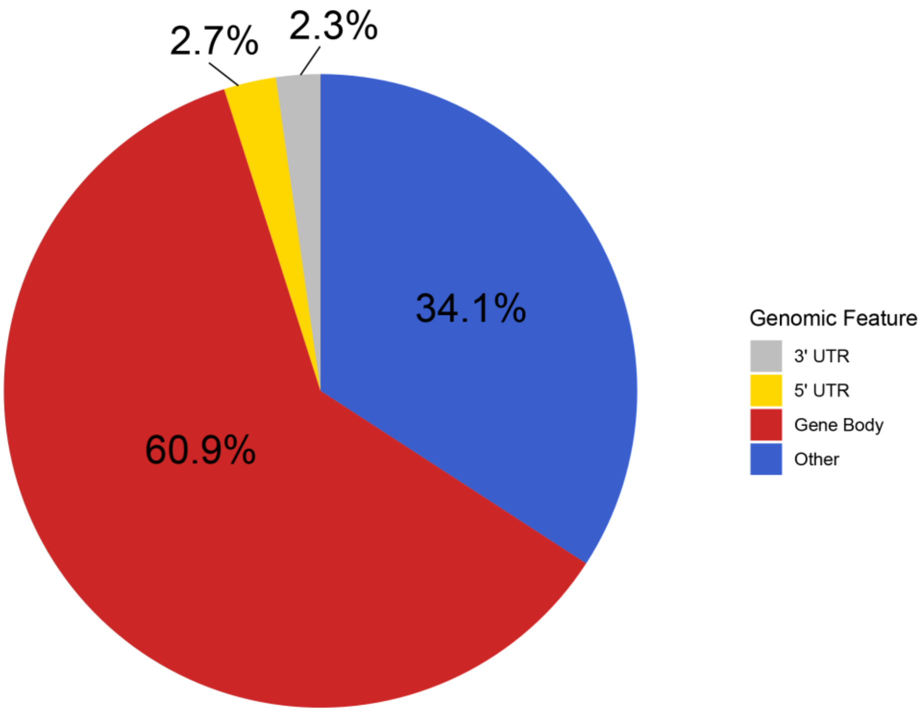

Supplementary Figure 1. Genomic distribution of CpG sites which were captured in reduced representation bisulfite sequencing.

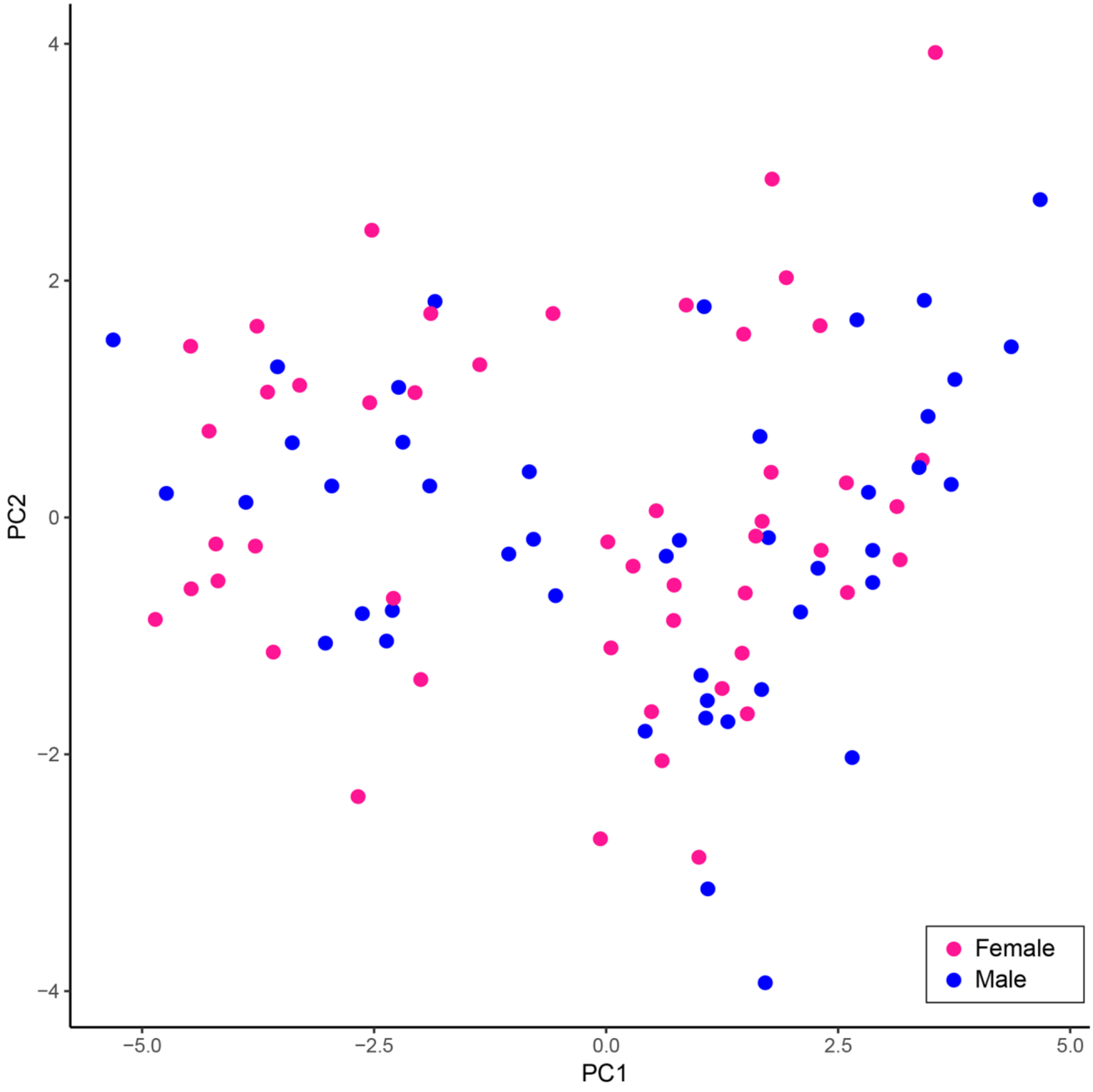

Supplementary Figure 2. Principle component analysis displaying no separation of sample sex.

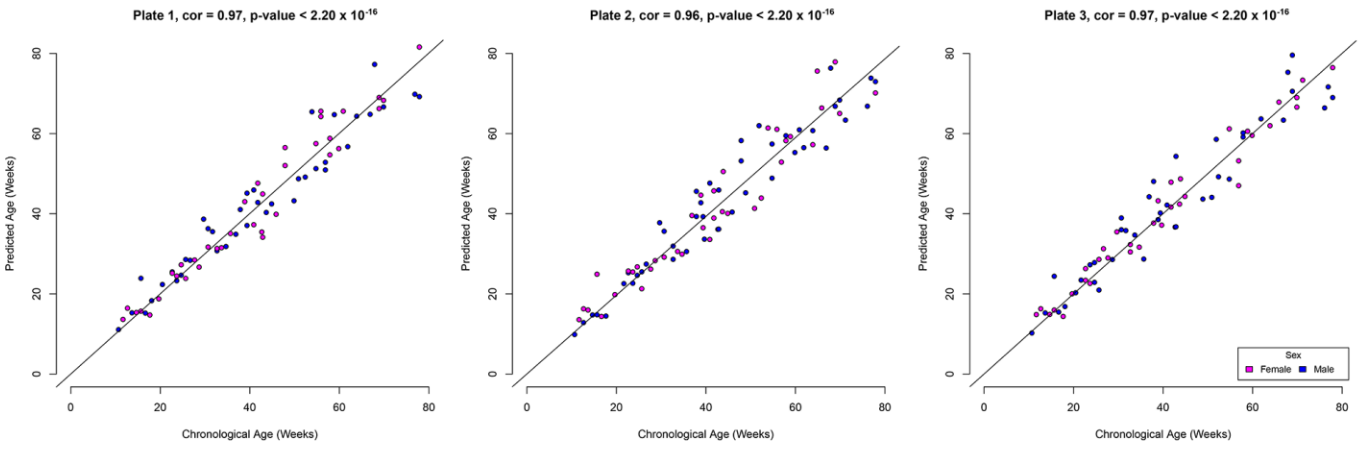

Supplementary Figure 3. Correlation between the chronological and predicted age in zebrafish by multiplex PCR. Samples were run in triplicate.

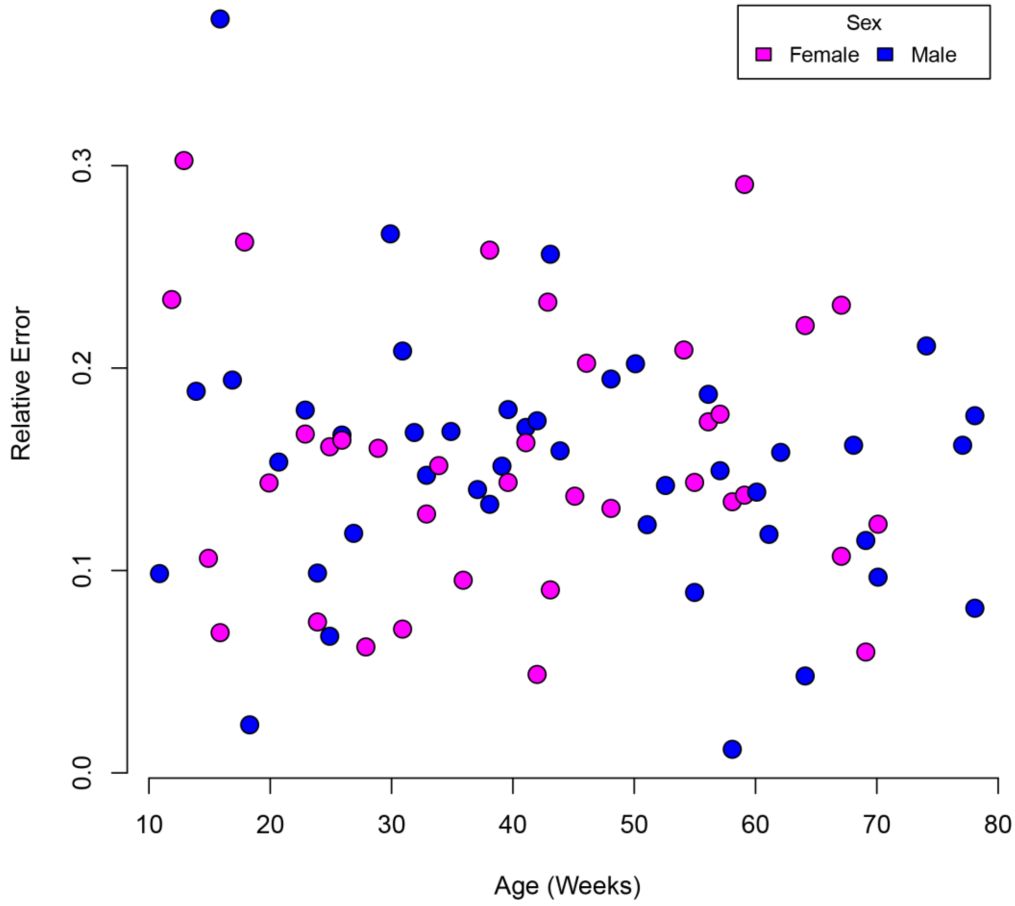

Supplementary Figure 4. Absolute error rate of samples by multiplex PCR over increasing age.

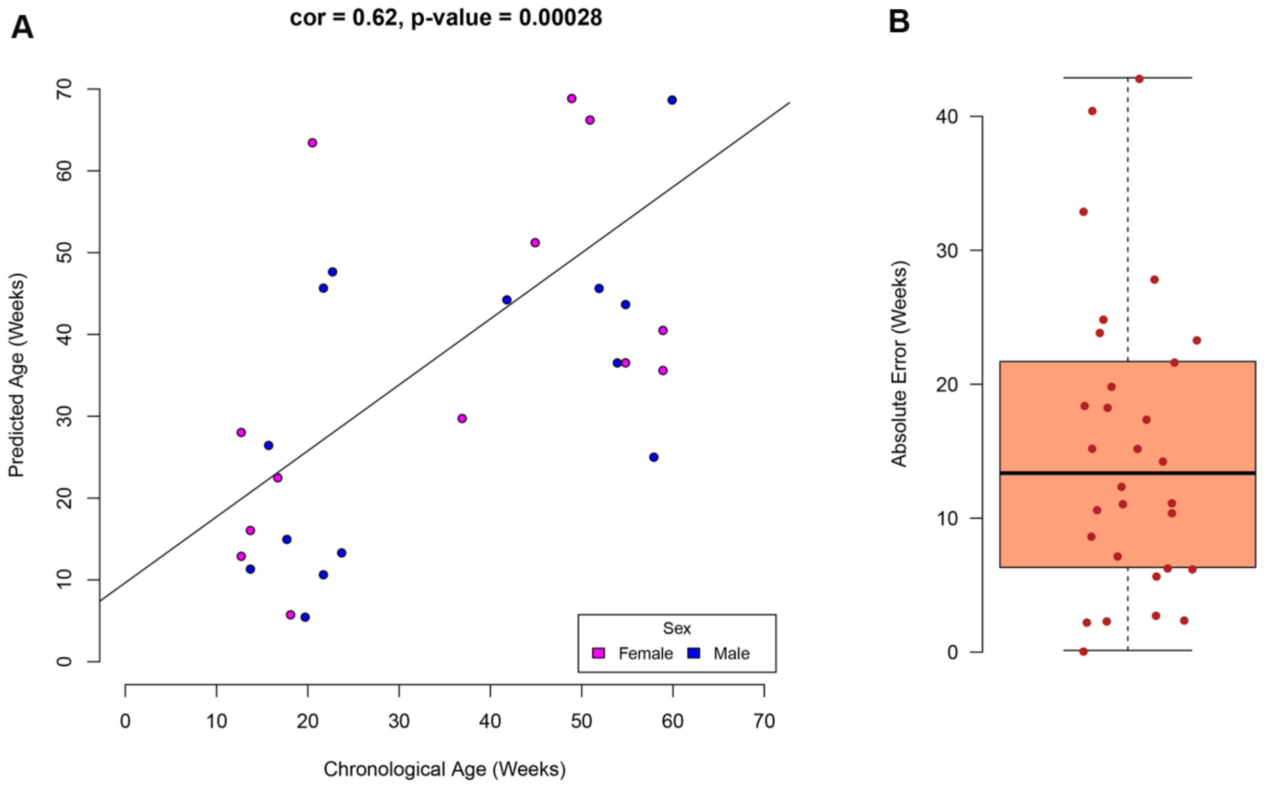

Supplementary Figure 5. Methylation-sensitive PCR to estimate age in zebrafish. (**A**) Correlation between the chronological and predicted age and (**B**) the absolute error rate in age estimation.
