# Supplementary material for: A DNA methylation age predictor for zebrafish
Source: Aging (Albany NY). 2020 Dec 23;12(24):24817–35. doi: 10.18632/aging.202400 (PMC7803548; doi:10.18632/aging.202400)
Supplement: Supplementary Table 2 [file aging-12-202400-s003.pdf]

SUPPLEMENTARY TABLE

Supplementary Table 2. Locations of the zebrafish clock sites used to estimate age using RRBS data and the closest genomic feature.

CpG site			Association with age			Closest feature				
chr	position	strand	Weight	Correlation	p-value	Gene	feature	start	end	strand
Intercept	NA	NA	3.261736	NA	NA	NA	NA	NA	NA	NA
chr12	21540399	+	-0.06868	-0.456308633	3.80E-06	mrpl27	exon	21563072	21563114	+
chr12	35432443	+	0.041155	0.425636828	1.90E-05	chmp6b	exon	35487001	35487160	+
chr13	31180246	+	0.422877	0.49406794	4.18E-07	mettl18	exon	31259863	31260037	+
chr13	38582448	+	0.287827	0.518416373	8.70E-08	zgc:153049	exon	38688631	38688754	+
chr14	38455793	-	-0.34896	-0.404759937	5.20E-05	csnk1a1	exon	38442661	38443287	-
chr14	45387151	+	-0.22242	-0.432519711	1.34E-05	sncb	exon	45619305	45619341	+
chr17	52836692	+	0.089225	0.44142766	8.45E-06	meis2a	exon	52833657	52835083	+
chr18	38107080	+	-0.40695	-0.407236996	4.63E-05	nucb2b	exon	38210387	38210462	+
chr18	50792250	+	-0.3449	-0.434582509	1.21E-05	reln	CDS	50795737	50795848	+
chr19	20077224	+	0.013643	0.428542532	1.64E-05	hibadha	CDS	20079490	20079646	+
chr1	23386154	+	0.267495	0.462650352	2.67E-06	mab21l2	CDS	23385795	23386871	+
chr1	43259461	+	-0.28726	-0.419849369	2.53E-05	cabp2a	exon	43425989	43426036	+
chr20	16578711	-	0.007467	0.459510595	3.18E-06	ches1	CDS	16578582	16579053	-
chr20	21624045	+	0.310809	0.383202718	0.000138	jag2b	exon	21573904	21575945	+
chr20	26523373	+	0.436491	0.468930078	1.87E-06	zbtb2b	exon	26504936	26508142	+
chr20	28928268	+	0.050606	0.492313214	4.66E-07	fntb	exon	28924424	28924877	+
chr21	23231786	+	-0.09242	-0.406502544	4.79E-05	alg8	exon	22864361	22864801	+
chr21	25150743	+	-0.33385	-0.541055377	1.80E-08	sycn.2	exon	25189953	25190586	+
chr24	19868851	+	-0.22858	-0.559326016	4.64E-09	LOC100334155	exon	20073262	20073368	+
chr24	4215673	+	0.06477	0.410284892	4.01E-05	wdr37	exon	3494784	3495510	+
chr25	14631230	+	0.217506	0.420374681	2.46E-05	mpped2	CDS	14637373	14637488	+
chr25	16313450	+	0.307822	0.482149108	8.63E-07	tead1a	CDS	16315617	16315681	+
chr25	36872756	+	-0.17805	-0.360931599	0.000352	chmp1a	exon	36871083	36871567	+
chr25	6461988	+	0.453596	0.408996487	4.26E-05	snx33	exon	6351734	6353787	+
chr2	8207957	+	0.258846	0.462933479	2.63E-06	chst2a	exon	8314444	8316603	+
chr3	23616782	+	-0.27465	-0.451308167	4.99E-06	hoxb3a	exon	23616752	23617534	+
chr4	17690807	+	-0.26411	-0.603855727	1.17E-10	gnptab	exon	17690788	17690922	+
chr4	18675145	+	-0.20748	-0.461522112	2.84E-06	slc26a4	CDS	18793563	18793599	+
chr5	51679905	+	0.034253	0.386666431	0.000118	slc14a2	exon	51529758	51531231	+
