# Supplementary material for: A DNA methylation age predictor for zebrafish
Source: Aging (Albany NY). 2020 Dec 23;12(24):24817–35. doi: 10.18632/aging.202400 (PMC7803548; doi:10.18632/aging.202400)
Supplement: Supplementary File 1 [file aging-12-202400-s006.docx]

Additional Methods to ‘A DNA methylation age predictor for zebrafish’

Benjamin Mayne^1,*^, Darren Korbie^2^, Lisa Kenchington^3^, Ben Ezzy^3^, Oliver Berry^1^ and Simon Jarman^4^

^1^ Environomics Future Science Platform, Indian Oceans Marine Research Centre, Commonwealth Scientific and Industrial Research Organisation (CSIRO), Crawley, Western Australia, Australia

^2^ Australian Institute for Bioengineering and Nanotechnology, The University of Queensland

Brisbane, Australia

^3^ Western Australian Zebrafish Experimental Research Centre (WAZERC), University of Western Australia, Perth, Western Australia, Australia

^4^ School of Biological Sciences, University of Western Australia, 35 Stirling Highway, Perth, Western Australia, Australia

* Author correspondence: [benjamin.mayne@csiro.au](mailto:benjamin.mayne@csiro.au)

The zebrafish were housed in the Western Australia Zebrafish Experimental Research Centre, an AAALAC accredited facility. The tanks were 3.5 L with a maximum housing density was 8 fish per L as a recirculating system (ZEBTEC Centralized Aquatic System, Tecniplast, Milan), with a water exchange rate (system volume) of 10% per 24 h and water exchange within tanks 9 times per hour. The municipal water supply was processed through a reverse osmosis system and activated carbon (100 microns), rotating drum (42 microns), biological (fluidized bed with biochips) and UV (200 000 uWs/cm^2^) filtration. Rooms are maintained at ~ 26 °C with a 14:10 h light dark cycle. The Tecniplast system automatically doses for conductivity, 800 µS +/- 100 µS (salts – Aquarium Systems Instant Ocean Aquarium Salt, France), pH 7.4 +/- 0.6 (sodium bicarbonate) and heating elements for the water temperature, with a set point 28.5 °C +/- 1.5 °C. System water is tested weekly using a water testing kit (API Liquid Test Kits, API) for general hardness (GH, 71.6 mg/L - 89.5 mg/L), carbonate hardness (KH, 17.9 mg/L – 35.8 mg/L), ammonia (<0.05 mg/L)_,_ nitrates (<100 mg/L) and nitrites (target 0 mg/L) and a colorimeter (DR 900, HACH) for pH, ammonia (<0.05 mg/L)_,_ nitrates (<100 mg/L) and nitrites (target 0 mg/L) and oxygen (>6 mg/L).

The health testing regime for the zebrafish colony involved testing of whole fish housed both pre- and post-filtration twice a year. *Edwardsiella ictaluri*, *Flavobacterium columnare*, *Ichthyophthirius multifiliis*, Infectious spleen& kidney necrosis virus (ISKNV), *Mycobacterium abscessus*, *Mycobacterium chelonae*, *Mycobacterium fortuitum*, *Mycobacterium haemophilum*, *Mycobacterium marinum*, *Mycobacterium peregrinum*, *Mycobacterium* spp., *Myxidium streisingeri*, *Piscinoodinium pillulare*, *Pleistophora hyphessobryconis*, *Pseudocapillaria tomentosa* and *Pseudoloma neurophilia* were not detected by PCR in fish samples*. Mycobacterium abscessus*, *Mycobacterium chelonae*, *Mycobacterium fortuitum*, *Mycobacterium haemophilum*, *Mycobacterium marinum*, *Mycobacterium peregrinum*, *Mycobacterium* spp., *Myxidium streisingeri*, *Pseudocapillaria tomentosa* and *Pseudoloma neurophilia* were not found by PCR in mulm or rotifer samples. The general health of the zebrafish was checked twice daily Monday to Friday and once daily on the weekends. These checked include visual observation of each cage for cleanliness, water flow and normal fish behaviour and general appearance. The fish were manually fed twice daily with a commercial pellet (two clicks of 70 mg each morning and one click of 70 mg in the afternoon, NRD 300-500µm, NRD, Thailand) and daily with Brachionus plicatilis (L-type) enriched roti grow plus (one squirt [5-10 mL] from a bottle of 200-400 rotifers/mL).

Buffered MS-222 was prepared in a biological safety cabinet with the operator wearing gloves, gown and a respirator. The concentration of buffered MS-222 was 168 mg/L from a stock solution of 8.4 g/L. The stock solution was created by dissolving 1.68 g of MS-222 (Ethyl 3-aminobenzoate methanesulfonate, Sigma Aldrich, China) in 200 mL of reverse osmosis water. Aliquots of this stock solution were wrapped in foil and frozen prior to use. The anaesthetic bath was made by adding 10 mL of thawed stock solution to 490 mL of tank water and gently dispersing the drug through the solution. The solution was then buffered using drops of 1 M sodium bicarbonate until the pH of the anaesthetic bath was around 7.0 -7.5.
